# Thanatophoric dysplasia in nonadherent to antenatal care in low middle income country: a rare case reports

**DOI:** 10.1097/MS9.0000000000001356

**Published:** 2023-10-04

**Authors:** Abhigan Babu Shrestha, Sanskriti Chapagain, Tungki Pratama Umar, Randhir Sagar Yadav, Shumneva Shrestha, Kiran Bhandari, Ranjana Sedai, Aryan Poudel, Chadani Mahat, Shreeya Sharma, Alish Bhandari

**Affiliations:** a Faculty of Internal Medicine, M. Abdur Rahim Medical College, Dinajpur; b Rangpur Community Medical College, Bangladesh; c Faculty of Medicine, Sriwijaya University, Palenbang, Indonesia; d University of Florida, Jacksonville, Florida, USA; e Maharajgunj Medical Campus, Institute of Medicine, Tribhuvan University, Kathmandu; f Lumbini Medical College, Palpa; g Devdaha Medical College and Research Institute, Bhaluhi, Rupandehi; h Nepal Army Institute of Health Sciences, Kathmandu, Nepal

**Keywords:** birth defects, micromelia, skeletal dysplasia, thanatophoric dysplasia

## Abstract

**Introduction and importance:**

Thanatophoric dysplasia is a rare, fatal, and sporadic form of skeletal dysplasia caused by a mutation in fibroblast growth factor receptor 3 (FGFR3). It is characterized by a conical thorax, platyspondyly (flat vertebral bodies), and macrocephaly. This disorder can be diagnosed antenatally as early as 13 weeks of gestation.

**Case presentation:**

The authors reported a case of thanatophoric dysplasia on USG in a 19 year old young consanguineous female in her second trimester of pregnancy. Ultrasound examination showed a clover leaf-shaped skull, a widened anterior fontanel, a coarse and edematous face, a flattened nasal bridge, a short neck, a low set of ears, shortening of both upper and lower limbs with short fingers, bowed thighs and legs, and a relatively narrow thorax.

**Clinical discussion:**

Lung hypoplasia, polyhydramnios, and hydrops in affected individuals lead to a poor prognosis. Hence, timely intervention should be done to avoid a poor prognosis. However, a mix of sonographic, genetic, histological, and autopsy studies are applied to make the most accurate diagnosis.

**Conclusion:**

The authors reported this case due to the rarity of this condition and the need for a systematic and multidisciplinary approach.

## Introduction

Highlights

- Thanatophoric dysplasia is the most fatal and rare disease characterized by micromelia, a small conical thorax, platyspondyly (flat vertebral bodies), and macrocephaly.
- It can be diagnosed antenatally on ultrasonography as early as 13 weeks of gestation as femoral length can be assessed.
- There is a need for a systematic and multidisciplinary approach to the diagnosis of thanatophoric dysplasia.

Thanatophoric dysplasia (TD) is a rare, congenital, sporadic, and lethal skeletal dysplasia caused by de novo autosomal dominant mutation in the fibroblast growth factor receptor 3 (FGFR3) gene, located on chromosome 4p16.3. It is characterized by micromelia, a small conical thorax, platyspondyly (flat vertebral bodies), and macrocephaly^1,2^. The estimated incidence of TD is 1 in 20 000 to 1 in 50 000 live births[1]. Various diagnostic techniques, such as sonography, genetics, histology, and autopsies, have been utilized to diagnose TD. Molecular analysis of FGFR3 gene mutations can be performed using fetal cells obtained through procedures like amniocentesis (15–18 weeks of pregnancy) or chorionic villous sampling (10–12 weeks of pregnancy)^3^.

It can also be diagnosed antenatally on ultrasonography (USG) as early as 13 weeks of gestation as femoral length can be assessed^4^. Hence, a comprehensive toolkit along with timely intervention is crucial for early detection and improving our understanding of the condition. Herein, we describe the case of TD type 2 with its significant clinical, and radiological features and various approaches for its diagnosis. The work has been reported in line with the Surgical CAse REport (SCARE) criteria^5^.

## Case presentation

**Figure 1:**
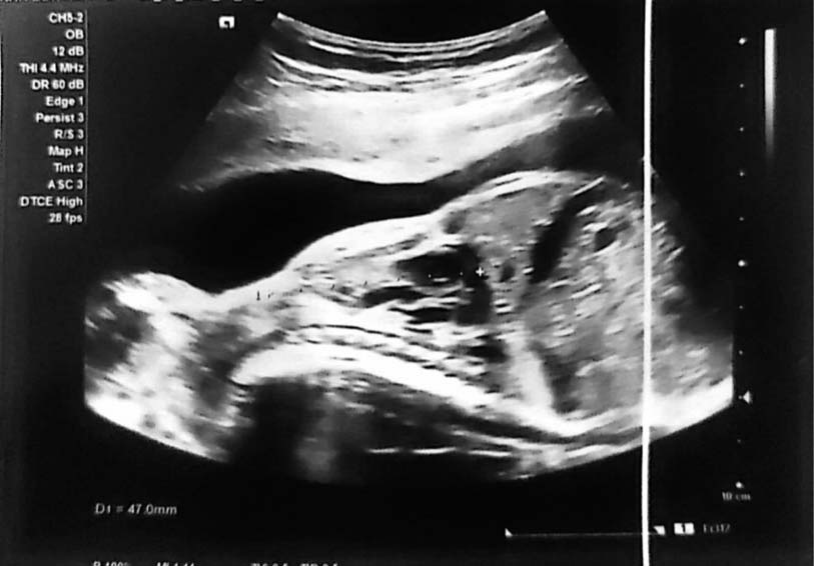
Ultrasonographic result of uterus showing clover-shaped skull, narrowed chest with short horizontal ribs, shortening of all the limbs.

A 19-year-old young female, primigravida presented to the gynecology and obstetric ward for routine antenatal care at 26 weeks of gestation. The patient was an unbooked case with no prior history of antenatal examination in her present pregnancy. There was no history of fever, rashes, spotting per vaginum, drug intake, or radiation exposure during the pregnancy. There was no family history and psychosocial history of congenital abnormalities, diabetes mellitus, hypertension, thyroid dysfunction, tuberculosis, etc. There was no history of alcohol consumption, smoking, or recreational drug use. The patient has been in consanguineous marriage for the past 4 years. Her vital and general physical examinations are within normal limits. On abdominal examination, symphysis fundal height was 26 weeks, with a longitudinal lie and cephalic presentation, palpated just above the pubic symphysis, and with a regular fetal heart rate of 144 bpm, suggesting a viable pregnancy. No abnormality was detected on respiratory, cardiovascular, or central nervous system examinations. The differential diagnosis of TD includes homozygous achondroplasia, achondrogenesis, campomelic dwarfism, rhizomelic chondrodysplasia punctata, short rib polydactyly syndromes, severe hypophosphatasia, and severe osteogenesis imperfecta. Blood investigations were within normal limits. On further evaluation, USG revealed a single live intrauterine fetus of 26 weeks and 3 days of gestational age with fetal heart rate 144 bpm, estimated fetal weight (EFW) 441 g, a biparietal diameter of 6.7 cm, a head circumference of 23.1 cm, a femoral length of 4.7 cm, an abdominal circumference of 19.6 cm, a humerus length of 3.9 cm. Shortening of bilateral hindlimbs and forelimbs, narrowed chest with short horizontal ribs, atrial septal defect, abnormal shape of skull giving the appearance of a cloverleaf skull, not clearly visible kidneys, no frontal bossing, and cleft palate are found as shown in the Figure 1. The amniotic fluid index was within the normal range.

**Figure 2:**
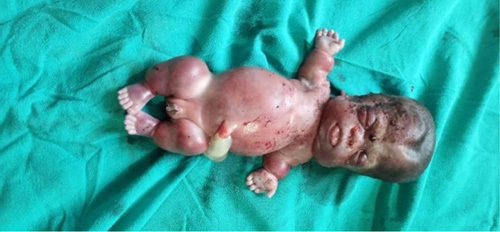
Thanatophoric dysplasia showing widened anterior fontanel, a coarse and edematous face, a flattened nasal bridge, a short neck, a low set of ears, shortening of both upper and lower limbs with short fingers, protruded abdomen.

Based on the above finding, severe skeletal dysplasia was diagnosed. The mother was counseled in detail by the obstetrician about diagnosis, prognosis, complications, and the termination of pregnancy. Induction of labor was recommended to the patient due to fetal congenital abnormalities. The procedure of induction of labor was performed by the obstetrician. The patient received misoprostol at a dose of 50 mg per vaginum in the posterior fornix, with the administration initiated at 4 am. Then the patient was placed in the left lateral position for a period of 30 min. Subsequently, regular reassessment was conducted every 4 h to monitor the progress of cervical changes. The initial assessment using the Bishop score indicated greater than 6, along with a cervical dilation greater than 2 cm and a cervical effacement of less than or equal to 30%. In response to these findings, the labor was augmented by introducing oxytocin. The patient delivered a preterm male baby, which did not cry after birth even after stimulation and suction, was admitted to the neonatal intensive care unit (NICU) immediately with respiratory distress, and expired after 1 h. The baby looks dysmorphic, with a widened anterior and posterior fontanel, an enlarged head, a coarse and edematous face, a flattened nasal bridge, a short neck, a low set of ears, shortening of both upper and lower limbs with short fingers, bowed thighs and legs, a relatively narrow thorax, and abdominal distension as shown in Figure 2. On parental evaluation, no apparent skeletal abnormalities were observed. The prenatal ultrasound scan findings, clinical findings, postmortem findings, and autopsy findings were consistent with severe skeletal dysplasia.

Due to the patient being lost to follow-up, intervention adherence, tolerability, and essential postintervention considerations such as ongoing monitoring and support were not effectively implemented.

## Clinical discussion

Mutations in the FGFR3 gene result in the receptor’s activation in the absence of growth factors, leading to the premature exit of proliferative cells from the cell cycle and their differentiation into prehypertrophic chondrocytes. This abnormal differentiation of chondrocytes contributes to the defective development of long bones^2,6^. Type 1 TD can be distinguished from type 2 TD by the presence of curved long bones that resemble a table telephone handle. A clover leaf-shaped head, straight long bones, and less extreme flatness of the vertebral bodies are characteristics of type 2 TD^7,8^.

Several other case reports have documented similar clinical features to those observed in our case^9^. Consanguinity in the family was mentioned in this case report, raising the likelihood of autosomal recessive inheritance^10^. Recently, biallelic loss of function of DNA replication fork stabilization factor (DONSON) has been linked to a severe form of microcephalic dwarfism called microcephaly-melia syndrome (MIMIS)^11,12^. Fetuses with this condition have significant growth restrictions, microcephaly, and a variety of limb deformities, which can cause intrauterine or perinatal mortality. Donson offers additional proof that microcephalic dwarfism is frequently caused by genes that encode either part of the DNA replication machinery (replisome) or proteins involved in genome stability^12^.

Antenatal skeletal dysplasia can be detected with USG, which is readily accessible in many settings. Since the fetal skeleton develops at a relatively early stage in the prenatal period, with endochondral ossification (appendicular and axial skeleton) or membraneous ossification (calvarium, section of clavicle, and pubis), ultrasound is extremely helpful for prompt detection of skeletal disorders^13,14^. The process can be identified in the clavicle and jaw as early as 8 weeks, in the appendicular skeleton by 12 weeks (a crucial time to find micromelia), in the ileum and scapula by 12 weeks, and in the metacarpals and metatarsals by 12–16 weeks^13,15^. According to other studies, the average gestational age of identification across all dysplasias was 22.7 weeks. In contrast, the mean gestational age upon diagnosis of fatal dysplasias was 20.4 weeks^16^.

The overall accuracy of prenatal diagnosis of skeletal dysplasias with standard USG is around 40%^13^. To avoid misdiagnoses and erroneous information about the likelihood of recurrence and inadequate patient care professional clinical and radiologic assessment should be used to provide a final diagnosis in every case^13,17^. An assortment of two-dimensional and three-dimensional ultrasounds and the prenatal sonographic method help to determine abnormal skeletal characteristics and then analyze an array of findings to provide some proper differential diagnoses for providing optimal patient management (both fetal and maternal) and estimating recurrence risk^13,18,19^. Although it is challenging to do so in the remote area where the discussed case originated, a systematic and multidisciplinary approach is necessary when a mix of sonographic, genetic, histological, and autopsy studies are applied to make the most accurate diagnosis^19^. Due to both financial constraints and the occurrence of a new mutation in the FGFR3 gene, which typically arises in individuals without any family history of the condition, we opted not to pursue chromosomal analysis and DNA molecular testing for FGFR3. We may also give the mother immediate emotional support and counseling, address any medical concerns, and provide guidance on family planning options and future care if she has not lost to follow-up.

The problem arises when a woman who has previously given birth to a child with skeletal dysplasia requests antenatal evaluation in her future pregnancy or, more frequently, when a routine sonographic scan accidentally reveals a shortened, bent, or abnormal appendage^16^. Prenatal diagnosis requires an accurate estimation of the fetal prognosis since patients may choose. A thorough examination performed as part of prenatal care (ANC) is crucial to terminate the pregnancy or forgo surgical delivery of an unviable fetus^16,20^. The results of an USG examination can reveal lethality parameters, such as a chest-to-abdominal circumference ratio of less than 0.618, a chest circumference of fewer than 5% at the height of the four-chamber section of the heart (hypoplastic thorax), a femur length-to-abdominal circumference ratio of less than 0.16, or specific distinguishing features that were indicative of an established lethal condition^13,16,21,22^. Furthermore, other seldom documented symptoms related to death include heart problems, renal anomalies, lymphatic aberrations, and the need for intensive care for any issues that may occur^23^. In addition, depending on the examination interval, it is possible to find shortened long bones (first trimester), polyhydramnios, relative macrocephaly, ventriculomegaly, a tiny chest cavity with short ribs, a bowed femur, growth deficiencies, and brain abnormalities (second and third trimesters)^24^.

Although it is encouraged to find skeletal dysplasia during regular checkups, several circumstances like advanced maternal age, a history of an impacted child or relative, concerns of restricted intrauterine growth, maternal diabetes mellitus, and multiple gestations raise the possibility that this condition exists and necessitate additional consideration for an USG examination during ANC^16^. Skeletal dysplasias can be inherited as X-linked, autosomal dominant, or recessive diseases. They can also be caused by imprinting errors, somatic mosaicism, exposure to teratogens, or sporadic patterns^13^. Because many skeletal dysplasias are linked to a high probability of recurrence, it is crucial to advise families and their doctors to make plans about acquiring and conserving tissue and/or DNA^13,25^. Based on the discovery of the genetic errors causing osteochondrodysplasias, about 160 of the 350 illnesses like TD, osteogenesis imperfecta, Roberts syndrome, and Ellis-van Creveld syndrome have received widespread attention^16^. From the patient’s perspective, both the diagnosis of severe skeletal dysplasia and the induction of labor were skillfully performed by the obstetrician during her antenatal care. Early detection is crucial because the outcomes of prenatal-onset skeletal dysplasias are typically poor and connected with lethality either in utero or shortly after birth due to pulmonary insufficiency (related to hypoplastic lungs or brain stem compression) or concomitant visceral defects^13,26^.

## Conclusion

The ultrasonographic and morphologic characteristics shown here are consistent with severe skeletal dysplasia. A combination of sonographic, genetic, histological, and autopsy examinations is critical for the diagnosis of skeletal dysplasia.

## Ethical approval

This research does not involve patients and therefore does not require ethical approval.

## Consent

Written informed consent was obtained from the patient for publication of this case report and accompanying images. A copy of the written consent is available for review by the Editor-in-Chief of this journal on request.

## Sources of funding

The research described in this work was not sponsored by any external sources.

## Author contribution

All authors contributed equally in the manuscript. A.B.S.: supervisor.

## Conflicts of interest disclosure

The authors declare that they have no financial conflict of interest with regard to the content of this report.

## Registration of research studies

1. Name of the registry: not applicable.
2. Unique identifying number or registration ID: not applicable.
3. Hyperlink to your specific registration (must be publicly accessible and will be checked): not applicable.

## Guarantor

Abhigan Babu Shrestha.

## Provenance and peer review

Not commissioned, externally peer-reviewed.

## References

[1] Wilcox WR Tavormina PL Krakow D. Molecular, radiologic, and histopathologic correlations in thanatophoric dysplasia. Am J Med Genet 1998;78:274–281. 9677066 10.1002/(sici)1096-8628(19980707)78:3<274::aid-ajmg14>3.0.co;2-c

[2] Legeai-Mallet L Benoist-Lasselin C Munnich A. Overexpression of FGFR3, Stat1, Stat5 and p21Cip1 correlates with phenotypic severity and defective chondrocyte differentiation in FGFR3-related chondrodysplasias. Bone 2004;34:26–36. 14751560 10.1016/j.bone.2003.09.002

[3] Rousseau F el Ghouzzi V Delezoide AL. Missense FGFR3 mutations create cysteine residues in thanatophoric dwarfism type I (TD1). Hum Mol Genet 1996;5:509–512. 8845844 10.1093/hmg/5.4.509

[4] Pretorius DH Rumack CM Manco-Johnson ML. Specific skeletal dysplasias in utero: sonographic diagnosis. Radiology 1986;159:237–242. 3513248 10.1148/radiology.159.1.3513248

[5] Agha RA Franchi T Sohrabi C SCARE Group. The SCARE 2020 guideline: updating consensus Surgical CAse REport (SCARE) guidelines. Int J Surg 2020;84:226–230. 33181358 10.1016/j.ijsu.2020.10.034

[6] van Ravenswaaij-Arts CM Losekoot M. From gene to disease; achondroplasia and other skeletal dysplasias due to an activating mutation in the fibroblast growth factor. Ned Tijdschr Geneeskd 2001;145:1056–1059. 11414167

[7] Brodie SG Kitoh H Lipson M. Thanatophoric dysplasia type I with syndactyly. Am J Med Genet 1998;80:260–262. 9843049 10.1002/(sici)1096-8628(19981116)80:3<260::aid-ajmg15>3.0.co;2-s

[8] Naveen NS Murlimanju BV Kumar V. Thanatophoric dysplasia: a rare entity. Oman Med J 2011;26:196–197. 22043415 10.5001/omj.2011.47 PMC3191698

[9] Jahan U Sharma A Gupta N. Thanatophoric dysplasia: a case report. Int J Reprod, Contrac, Obstet Gynecol 2019;8:758–761.

[10] Al-Gazali LI Bakir M Hamid Z. Micromelic dwarfism–humerus, femur and tibia type. Clin Dysmorphol 2001;10:24–28. 11152143 10.1097/00019605-200101000-00005

[11] Schulz S Mensah MA de Vries H. Microcephaly, short stature, and limb abnormality disorder due to novel autosomal biallelic DONSON mutations in two German siblings. Eur J Hum Genet 2018;26:1282–1287. 29760432 10.1038/s41431-018-0128-0 PMC6117362

[12] Abdelrahman HA John A Ali BR. Further delineation of the microcephaly-micromelia syndrome associated with loss-of-function variants in DONSON. Mol Syndromol 2019;10:171–176. 31191207 10.1159/000497337 PMC6528082

[13] Krakow D Lachman RS Rimoin DL. Guidelines for the prenatal diagnosis of fetal skeletal dysplasias. Gen Med 2009;11:127–133. 10.1097/GIM.0b013e3181971ccb PMC2832320 19265753

[14] Olsen BR Reginato AM Wang W. Bone development. Annu Rev Cell Dev Biol 2000;16:191–220. 11031235 10.1146/annurev.cellbio.16.1.191

[15] van Zalen-Sprock RM Brons JT van Vugt JM. Ultrasonographic and radiologic visualization of the developing embryonic skeleton. Ultrasound Obstet Gynecol 1997;9:392–397. 9239824 10.1046/j.1469-0705.1997.09060392.x

[16] Parilla BV Leeth EA Kambich MP. Antenatal detection of skeletal dysplasias. J Ultrasound Med 2003;22:255–258; quiz 259-261. 12636325 10.7863/jum.2003.22.3.255

[17] Calder AD Foley P. Skeletal dysplasias: an overview. Paediatrics and Child Health 2018;28:84–92.

[18] de Souza Lima T Ferreira BG Loureiro Souza CW. Prenatal diagnosis of diastrophic dysplasia in the second trimester of pregnancy: two- and three- dimensional ultrasonographic findings. Turk J Obstet Gynecol 2021;18:258–263. 34581174 10.4274/tjod.galenos.2021.35033 PMC8480210

[19] Agarwal A Agarwal S. Fetal micromelia, thoracic dysplasia and polydactyly revisited: a case-based antenatal sonographic approach. Ultrasound 2019;27:196–201. 32549900 10.1177/1742271X19847223 PMC7273877

[20] Yolanda N Yulianto F Arina S. A full-term infant with type II thanatophoric dysplasia. Case Reports in Perinatal Medicine 2019;8. doi:10.1515/crpm-2018-0035

[21] Dighe M Fligner C Cheng E. Fetal skeletal dysplasia: an approach to diagnosis with illustrative cases. Radiographics 2008;28:1061–1077. 18635629 10.1148/rg.284075122

[22] Nelson DB Dashe JS McIntire DD. Fetal skeletal dysplasias: sonographic indices associated with adverse outcomes. J Ultrasound Med 2014;33:1085–1090. 24866616 10.7863/ultra.33.6.1085

[23] Milks KS Hill LM Hosseinzadeh K. Evaluating skeletal dysplasias on prenatal ultrasound: an emphasis on predicting lethality. Pediatr Radiol 2017;47:134–145. 27904917 10.1007/s00247-016-3725-5

[24] Jimah BB Mensah TA Ulzen-Appiah K. Prenatal diagnosis of skeletal dysplasia and review of the literature. Case Rep Obstet Gynecol 2021;2021:9940063. 33953997 10.1155/2021/9940063 PMC8057870

[25] Krakow D. Skeletal dysplasias. Clin Perinatol 2015;42:301–319. 26042906 10.1016/j.clp.2015.03.003 PMC4456691

[26] Krakow D Alanay Y Rimoin LP. Evaluation of prenatal-onset osteochondrodysplasias by ultrasonography: a retrospective and prospective analysis. Am J Med Genet A 2008;146A:1917–1924. 18627037 10.1002/ajmg.a.32269 PMC2713784

